# Publicly Available, Interactive Web-Based Tools to Support Advance Care Planning: Systematic Review

**DOI:** 10.2196/33320

**Published:** 2022-04-20

**Authors:** Charlèss Dupont, Tinne Smets, Fanny Monnet, Lara Pivodic, Aline De Vleminck, Chantal Van Audenhove, Lieve Van den Block

**Affiliations:** 1 End-of-Life Care Research Group Vrije Universiteit Brussel & Ghent University Brussels Belgium; 2 Department of Family Medicine and Chronic Care Vrije Universiteit Brussel Brussel Belgium; 3 LUCAS Center for Care Research and Consultancy KU Leuven Leuven Belgium

**Keywords:** advance care planning, systematic review, web-based tools, health communication, quality of online content

## Abstract

**Background:**

There is an increasing number of interactive web-based advance care planning (ACP) support tools, which are web-based aids in any format encouraging reflection, communication, and processing of publicly available information, most of which cannot be found in the peer-reviewed literature.

**Objective:**

This study aims to conduct a systematic review of web-based ACP support tools to describe the characteristics, readability, and quality of content and investigate whether and how they are evaluated.

**Methods:**

We systematically searched the web-based gray literature databases OpenGrey, ClinicalTrials.gov, ProQuest, British Library, Grey Literature in the Netherlands, and Health Services Research Projects in Progress, as well as Google and app stores, and consulted experts using the following eligibility criteria: web-based, designed for the general population, accessible to everyone, interactive (encouraging reflection, communication, and processing of information), and in English or Dutch. The quality of content was evaluated using the *Quality Evaluation Scoring Tool* (score 0-28—a higher score indicates better quality). To synthesize the characteristics of the ACP tools, readability and quality of content, and whether and how they were evaluated, we used 4 data extraction tables.

**Results:**

A total of 30 tools met the eligibility criteria, including 15 (50%) websites, 10 (33%) web-based portals, 3 (10%) apps, and 2 (7%) with a combination of formats. Of the 30 tools, 24 (80%) mentioned a clear aim, including 7 (23%) that supported reflection or communication, 8 (27%) that supported people in making decisions, 7 (23%) that provided support to document decisions, and 2 (7%) that aimed to achieve all these aims. Of the 30 tools, 7 (23%) provided information on the development, all of which were developed in collaboration with health care professionals, and 3 (10%) with end users. Quality scores ranged between 11 and 28, with most of the lower-scoring tools not referring to information sources.

**Conclusions:**

A variety of ACP support tools are available on the web, varying in the quality of content. In the future, users should be involved in the development process of ACP support tools, and the content should be substantiated by scientific evidence.

**Trial Registration:**

PROSPERO CRD42020184112; https://tinyurl.com/mruf8b43

## Introduction

Via a European consensus process, advance care planning (ACP) has been defined as a process that enables individuals to define goals and preferences for future medical care, discuss these preferences with family and health care providers, and record these preferences and choices [1,2]. In recent decades, the concept of ACP has changed considerably, shifting from a clinician-led and documentation-focused process that emphasizes the need for advance directives to a broader concept of ongoing communication regarding various aspects of future care and treatment planning [3,4]. In the recent public health literature, the concept has been broadened further by emphasizing the opportunities that ACP conversations offer for normalizing and reshaping how we think, talk, and make decisions about the last chapters of our lives [5,6].

To support people in having such conversations, a wide variety of ACP support tools have been developed in several formats such as print or websites. They exist in different kinds of modalities (guides, card games, and videos) and for different target groups: people with specific diseases and their families, family caregivers, or the general public [7,8]. With the growing use of the internet and international efforts to promote ACP [9], more web-based ACP support tools are becoming publicly available [10]. An advantage of these web-based tools is that they are easily accessible to a large audience, are often interactive, and can thus be tailored to the needs of the user. Interactive elements include, for example, questions or exercises to encourage reflection and communication and process the information provided [10-12].

A recent review of the published peer-reviewed studies assessed the feasibility and effectiveness of interactive web-based ACP support tools for adult patients, relatives, and healthy individuals and found that users considered the tools easy to use and not burdensome. It also demonstrated that they could improve a user’s knowledge of ACP, ACP communication with relatives and health care professionals, and ACP documentation [8]. However, this review was not able to include all ACP support tools available to the general public, as many exist only on the web and have not been published in academic journals [7,8]. Reviewing these web-based tools is important as the quality of web-based content can vary widely or be based on personal opinions and experiences rather than on scientific evidence [13-16]. This can be particularly problematic with regard to ACP, as the content may be biased in favor of or against certain medical interventions [15], whereas the primary purpose of ACP should be to promote choices based on individual values and preferences [1,2].

Currently, there is no comprehensive overview of interactive ACP tools available on the internet. Therefore, this systematic review aims to answer the following research questions:

1. What are the characteristics and functionalities of interactive web-based ACP support tools?
2. How is ACP addressed in these tools?
3. What is the readability and quality of their content, and have they been evaluated in a study, and if so, what is their level of evidence?

## Methods

### Review Design and Protocol Registration

We conducted a systematic review of web-based interactive ACP support tools (hereafter called tools) following the 4 search strategies for web-based gray literature by Godin et al [17]. Search, selection, and data synthesis were performed between September 2020 and January 2021. The protocol for this systematic review was registered with PROSPERO (International Prospective Register of Systematic Reviews; CRD42020184112).

### Eligibility Criteria

We searched for tools that met the following inclusion criteria:

1. Designed to support the general population; that is, people with or without serious illnesses and their families
2. Available on the internet
3. Accessible to whoever visits the tool and can be used by anyone
4. Interactive; that is, encourage the user to reflect, communicate, formulate decisions, or document wishes [10-12]
5. In English or Dutch

Tools that exclusively aimed to support health care professionals in the ACP process were excluded.

### Search Strategies

We systematically searched for tools using the four search strategies recommended by Godin et al [17]: (1) web-based gray literature databases, (2) search engines, (3) app stores, and (4) expert consultation. The first three search strategies were conducted separately by 2 researchers (CD and FM), who both used the same search combinations (Multimedia Appendices 1-3) on different computers with *clean* (deleted cookies and history) browsers without being logged into a Google account. Because, as with peer-reviewed databases, every search database (gray literature databases, search engines, and app stores) has its own search functionalities and filters, we adapted the search terms to fit into the search fields of each database.

Furthermore, as search engines have their own algorithms for showing relevant results, we used several different search combinations (ie, combining the search terms, their permutations, and trending keywords) in search strategy 2 to minimize the risk of omitting relevant sources (Multimedia Appendix 2). As a search engine can give an overwhelming amount of *hits*, we screened the first 15 pages (first 150 hits) of each search combination.

We screened the available content of the results, such as executive summaries, the webpage *about*, or the explanation of the tool—when available—until we could answer the following question: *is this tool potentially a web-based ACP support tool for the general population*?. If the answer was *yes*, we included the tool for full screening and transferred the results to a Microsoft Excel file (with the name of the tool or website if there was no specific name for the tool).

For the first search strategy (gray literature databases), we used the following databases: *OpenGrey, ClinicalTrials.gov, ProQuest, British Library, Grey Literature in the Netherlands,* and *Health Services Research Projects in Progress*. For the second search strategy, we used the *Google search engine*, and for the search in app stores, we used *Google Play Store* and *Apple App Store.*

The fourth search strategy entailed consultation with experts on ACP. We identified experts via frequently listed and cited authors of the relevant literature, known stakeholders, and suggestions from other key informants. We consulted these experts by email and, to achieve saturation, asked them whether they knew any other tools that we had not found using the first three search strategies. The responses were added to the Microsoft Excel file and saved for the final selection.

### Selection of Web-Based ACP Support Tools

For the final selection, the 2 researchers (CD and FM) separately screened all available content of each potentially relevant tool against the eligibility criteria. In cases of disagreement about whether to include a tool, a third reviewer (TS) screened it and made the final decision on including it. We used Archive.is to archive the home page or the first page of the tools.

### Evaluation of the Readability and Quality of the Content of the Included Tools

Readability was evaluated using web-based readability analysis tools. These readability tools calculate several readability scores based on the characteristics of the text, such as the number of syllables per word and number of words per sentence. As the web-based readability analysis tools were exclusively for one language, we used two tools: one for the English tools [18] and one for the Dutch tools [19]. To determine readability, we used the Common European Framework of Reference for Languages (CEFR) levels [20,21]. The CEFR levels can be used to determine both English and Dutch readability and are calculated by the algorithm of web-based readability analysis tools and look at the number of words per sentence, number of pronouns and prepositions in a sentence, and number of simple words [22]. CEFR comprises six reading levels (A1, A2, B1, B2, C1, and C2), with A1 as the easiest level and C2 as the most difficult. The recommended readability standard for CEFR is B1 [22].

To evaluate the quality of the content of the tools, we used the validated *Quality Evaluation Scoring Tool* (QUEST) [23]*.* This quality assessment tool can be used to assess web-based health content by evaluating 7 items, each assigned with a weighted score. Six items have a possible score between 0 to 1 or 0 to 2 and weight between 1 and 3: authorship (score 0-2×1), conflicts of interest (score 0-2×3), complementarity (whether they support the patient-physician relationship; score 0-1×1), currency (if the content is frequently updated; score 0-2×1) and the tone of the content (whether the content was *fully supported* using strong vocabulary such as *cure*, *guarantee*, and *easy*; *mainly supported* where the authors mainly support their claims but with more cautious vocabulary; or *balanced/cautious support* with statements of limitations or contrasting findings; score 0-2×3). The seventh item is attribution (whether and what kind of sources are used to create the content) and is measured in two steps: first, identifying the presence of references to scientific studies (score 0-3×3) and, second, when scoring >1, identifying the type of studies referred to in vitro, observational studies, or meta-analyses or clinical trials (score 0-2×1) [23,24]*.* Each tool was evaluated for each of the 7 items. The scores for each item were summed to create a total quality score ranging between 0 and 28, with higher scores indicating a better quality of the content in the tool.

Readability was evaluated by 2 researchers (CD and FM) who copy-pasted the text of a full webpage into the text fields of the web-based readability score calculator tools. The same 2 researchers assessed the content of the tools to determine the QUEST score for each tool. Any disagreements on readability and QUEST scores were discussed to reach a consensus. If no consensus was found, a third researcher (TS) made the final decision.

### Evaluation of the Level of Evidence of the Included Tools

To assess whether the included tools had been evaluated as part of a research study, we (1) screened each one for any information on evaluation; (2) we searched the gray literature databases OpenGrey, ClinicalTrials.gov, ProQuest, British Library, Grey Literature in the Netherlands, and Health Services Research Projects in Progress; and (3) we searched for publications of primary peer-reviewed studies in PubMed, Web of Science, PsycINFO, and CINAHL. Search terms in the database included the names of the tools, websites, or persons or organizations involved in the development of the tool.

We screened the available content (for scientific article abstracts or, when needed, full texts) to check whether the result was a primary study on one of the included tools. If it was, where possible, we determined the level of evidence using the Hierarchy of Evidence from the National Health and Medical Research Council [25,26] to determine how the tools were evaluated.

Two researchers (CD and FM) independently assessed the full texts of the peer-reviewed studies to determine the level of evidence and assess what was evaluated (usability and effectiveness). Any disagreements were discussed to reach a consensus. If no consensus was reached, a third researcher (TS) made the final decision.

### Data Synthesis and Outcomes of Interest

To answer the research questions, we developed 4 data extraction tables. For the first 3 extraction tables, the 2 researchers (CD and FM) independently assessed the content of the tools to summarize the addressed characteristics, functionalities, and key elements of ACP. Any disagreements were discussed to reach a consensus. If no consensus was reached, a third researcher (TS) made the final decision.

The first extraction table was used to assess the aim; target group; available languages; format; and where, by whom, and how the tools were developed. We evaluated the used functionalities by developing a second extraction table based on a review of peer-reviewed studies by van der Smissen et al [8]. We slightly changed the 12 assessed functionalities by removing *can be used without assistance,* as in our review, we only included tools that were designed to be used by the general population, and we added the functionalities *predetermined path and possibility to save input and return* as these allow the user to conduct the ACP process at their own pace and *input can be printed* as this functionality can increase the accessibility of websites [27]. Therefore, in this review, we extracted the following data with regard to the functionalities of ACP tools:

1. The tool is free of charge.
2. Registration is needed.
3. There is a save and return option.
4. It is possible for the user to give input.
5. The tool is tailored to the user.
6. The tools provide feedback based on the input of the user.
7. The input from the user can be printed.
8. The tool suggests a predetermined ACP path.
9. The tool gives an indication of the progress of the user.
10. Videos are used, as well as hyperlinks to other web pages.
11. There is a text-to-speech option.
12. A privacy policy is mentioned.
13. Data log analysis is mentioned.

The manner in which ACP is addressed in the tools was extracted using 14 ACP key elements (the third extraction table) as an analytic framework. The 14 key elements were self-developed and based on the recommendations of Rietjens et al [1], the consensus definition of ACP of Sudore et al [2], and the review by van der Smissen et al [8]. From these definitions, we aimed to extract all relevant elements that can be part of the ACP; that is, the following elements:

1. Providing information on ACP
2. Providing information on legal frameworks
3. Address readiness and timing for ACP
4. Stimulates to explore personal values and goals
5. Stimulates to explore preferences regarding future care
6. Stimulates to explore uncertainties and consequences
7. Stimulates to explore preferences regarding the last days of life
8. Stimulates to explore preferences for a possible proxy decision-maker
9. Encourages to appoint a proxy decision-maker
10. Encourages to discuss ACP with family
11. Encourages to discuss ACP with health care professionals
12. Encourages to document ACP
13. Encourages to generate that document (in the tool)
14. Encourages to share that document.

The readability, quality of the content, information about the evaluation of the tools, and their level of evidence were summarized in the fourth extraction table.

## Results

### Selection and Inclusion of the Tools

**Figure 1:**
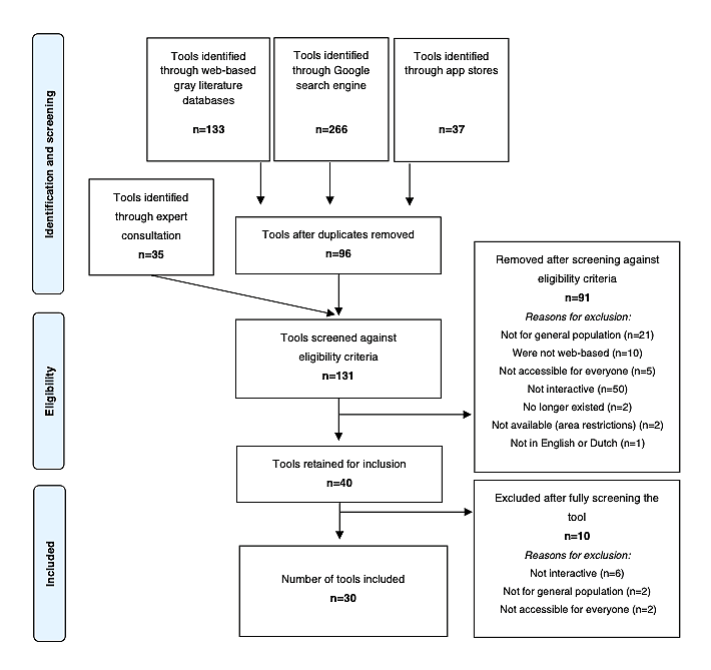
PRISMA (Preferred Reporting Item for Systematic Reviews and Meta-Analyses) flowchart of the selection process.

We found 436 tools using the first 3 search strategies. After removing duplicates (the tools retrieved and the removed duplicates per search strategy can be found in Multimedia Appendix 4), a list of 96 potential interactive web-based ACP support tools for the general population was sent to 15 experts on ACP. Of the 15 experts, 14 (93%) replied, and together they identified 35 additional tools. Two researchers (CD and FM) subsequently screened the 131 tools against the eligibility criteria. Approximately 69.5% (91/131) were excluded (Figure 1), and there were no conflicts to resolve regarding exclusion. The remaining 30.5% (40/131) of tools were fully screened (ie, all text available in the tool for data extraction), and we ended up excluding 10 more in an agreement between both researchers. Thus, the total number of included tools was 30. An overview of screening, selection, and reasons for exclusion and inclusion is shown in Figure 1.

### Characteristics of the Tools

Approximately 80% (24/30) of tools targeted the general population, although 20% (6/30) also aimed to target health care professionals (Multimedia Appendix 5 [28-57]). For instance, the app *ACDCare* could be used by patients to make an advance care directive that can be uploaded to their health record and to which the health care professional has access [28]. The tools *Mydirectives, Be My Voice, My Living Will, Speak Up,* and *Considering your own future health care* not only supported the general population in ACP but also provided information on ACP for health care professionals [29-33]. Of the 30 tools, 24 (80%) did mention a clear aim; 7 (23%) supported reflection or communication, 8 (27%) supported people in making decisions, 7 (23%) provided support to document decisions, and 2 (7%) aimed to achieve all these objectives.

Of the 30 tools, 15 (50%) were websites, 10 (33%) were web-based portals with the possibility of logging in and returning to personal information, 3 (10%) were apps, and 2 (7%) had a combination of formats. For example, *PREPARE for Your Care* (*PREPARE*) can be used via the website or by logging in to a web-based portal [34].

Of the 30 tools, 12 (40%) were developed in the United States, 6 (20%) in Canada, 4 (13%) in Australia, 3 (10%) in the Netherlands, 3 (10%) in the United Kingdom, 1 (3%) in New Zealand, and 1 (3%) in Belgium. Approximately 83% (25/30) were owned by nonprofit organizations. Approximately 23% (7/30) provided information on their development; all were developed in collaboration with health care professionals, and 10% (3/30) were also developed with end users.

### Functionalities of the Tools

Of the 30 tools, 27 (90%) were free of charge, and 17 (57%) were available without registration (Multimedia Appendix 6 [28-57]). Approximately 97% (29/30) of tools offered users the option of providing input by responding to a question or statement, for example, by asking them to write in an empty box. With regard to the flow of the tool (the steps or path a user must take to go through it), 97% (29/30) used a predetermined path [28-44,46-57]. For example, if there are *x* number of steps in a tool, the user needs to go through these steps to *finish* the ACP process provided in the tool. One of the tools (the *Go Wish* card game) does not have a set path but uses a mechanism in which users can sort cards on particular wishes and preferences to stimulate reflection and communication [45]. Approximately 17% (5/30) used the input of the user to tailor information, redirect them to a specific webpage with more information, or ask for clarification on the input. For example, in *My Values,* when identifying *not wanting to be a burden* as important, a screen pops up asking them to explain briefly (by typing) what *becoming a burden* means to them [49]. One also uses input to give feedback (Multimedia Appendix 6 [28-57])—*PREPARE* not only provides information based on input but also provides tips when, for example, the user indicates *not ready to choose a proxy decision-maker* [34].

### ACP Elements Addressed in the Tools

All tools contained at least seven of the 14 ACP key elements identified in our analytical framework, and 20% (6/30) of tools comprised all (Multimedia Appendix 7 [28-57]). With regard to the information available, 40% (12/30) provided both information on ACP and on the legal frameworks of ACP. Of the 30 tools, readiness and timing of ACP were addressed in 15 (50%), encouraging people to explore personal values and goals in 28 (93%), and preferences regarding future care in 29 (97%) tools; uncertainties and consequences of hypothetical serious illness scenarios were addressed in 25 (83%), preferences regarding the last days of life in 27 (90%), and the possibility of appointing a proxy decision-maker in 21 (70%) tools. Of the 30 tools, 20 (67%) encouraged appointing a proxy decision-maker, and 27 (90%) encouraged discussing ACP with family or a health care professional and documenting ACP outcomes (for example, using an advance directive); in 27 (90%) tools, it was possible to document one’s wishes and preferences within the tool itself and share this document with others (by printing, via email, or via a direct link; Multimedia Appendix 7 [28-57]).

### Readability and Quality of the Content

The readability of the ACP tools varied (Table 1); however, 83% (25/30) of ACP tools had a B1 or lower CEFR level. The QUEST scores of the tools varied between 11 and 28 (theoretical scale scores between 0 and 28; Table 2). Most of the lower-scoring tools did not refer to any sources to support the information they contained, were not current (ie, not updated in the past 5 years), or did not provide any information on authorship. The tools that scored ≥21 used at least one reference to a scientific study to support the information in the tool.

**Table 1:** Readability scores of the interactive, web-based ACP^a^ support tools.^b^

Tools	CEFR^c^ scale
My decisions	A1
My living voice	A1
Speak up	A1
PREPARE^d^	A2
Go Wish card game	A2
MyDirectives	A2
Plan Your Life Span	A2
Lets Think Ahead—My ACP	A2
Advance Care Planning: Should I Stop Treatment That Prolongs My Life?	B1
The Letter project Advance Directive	B1
Advance Care Planning: Should I Receive CPR^e^ and Life Support?	B1
MyWishes	B1
My Living Will	B1
Everplans	B1
Considering your own future health care	B1
Tijdig nadenken over het levenseinde	B1
Oog in Oog	B1
Verken uw wensen voor zorg en behandeling	B1
Dying to Talk	B1
Be my voice	B1
ACDCare	B1
Five Wishes	B1
MyValues	B1
Dementia Values and Priorities Tool	B1
Advance Care Planning: Should I Have Artificial Hydration and Nutrition?	B2
Planning for Your Future	B2
Beslishulp—Vroegtijdige zorgplanning	B2
Cake	B2
NVLivingWill	B2
Advance Care Planning: Should I Stop Kidney Dialysis?	C1

^a^ACP: advance care planning.

^b^Ranked from lowest (A1) to highest score (C2) possible.

^c^CEFR: Common European Framework of Reference for Languages.

^d^PREPARE: PREPARE for Your Care.

^e^CPR: cardiopulmonary resuscitation.

**Table 2:** QUEST^a^ scores of the interactive, web-based ACP^b^ support tools.^c^

Tools	QUEST score
PREPARE^d^	28
Go Wish card game	23
Considering your own future health care	20
Everplans	20
Oog in Oog	20
Dementia Values and Priorities Tool	19
Dying to Talk	19
My Living Will	19
MyDirectives	19
NVLivingWill	19
Plan your Life Span	19
Cake	17
Five Wishes	17
Lets Think Ahead–My ACP	17
Planning for Your Future	17
Be my voice	16
Beslishulp-Vroegtijdige zorgplanning	16
MyValues	16
MyWishes	16
My decisions	14
Advance Care Planning: Should I Have Artificial Hydration and Nutrition?	13
Advance Care Planning: Should I Receive CPR^e^ and Life Support?	13
Advance Care Planning: Should I Stop Kidney Dialysis?	13
Advance Care Planning: Should I Stop Treatment That Prolongs My Life?	13
My living voice	12
ACDCare	11

^a^QUEST: Quality Evaluation Scoring Tool.

^b^ACP: advance care planning.

^c^Ranked from lowest (0) to highest score (28) possible.

^d^PREPARE: PREPARE for Your Care.

^e^CPR: cardiopulmonary resuscitation.

### Evaluated Tools and Their Level of Evidence

Of the 30 included tools, 5 (16%) tools had been evaluated in a study, all of which were published in the peer-reviewed literature (Table 3). *Verken uw wensen voor zorg en behandeling* [57] is under evaluation; however, the results have not yet been published. *MyDirectives* and *NVLivingWill* were studied using a posttest design [58-60], and *PREPARE, Plan Your Life Span*, and *The Letter project Advance Directive* were studied in at least one properly designed randomized controlled trial [61-71]. The study on *NVLivingWill* evaluated the ease of use of the tool [58]; the studies on *PREPARE* evaluated ease of use, effectiveness, acceptability, and understandability (information in the tool was easy to read) [63-71]; *Plan Your Life Span* evaluated effectiveness [61]; and the study on *The Letter project Advance Directive* evaluated understandability [72].

**Table 3:** Level of evidence of the interactive, web-based advance care planning support tools.

Tool, authors, and title of the study				The hierarchy of evidence^a^
***MyDirectives* [29]**				Evidence obtained from a posttest
	Holland et al [59]	Nurse-led patient-centered advance care planning in primary care		
	Fine et al [60]	Early experience with digital advance care planning and directives, a novel consumer-driven program		
***NVLivingWill* [51]**				Evidence obtained from a posttest
	Klugman and Usatine [58]	An evaluation of 2 online advance directive programs		
***Plan Your Life Span* [53]**				Evidence obtained from at least one properly designed randomized controlled trial
	Ramirez-Zohfeld et al [62]	Longitudinal follow-up of long-term care planning using PlanYourLifespan.org		
	Lindquist et al [61]	PlanYourLifeSpan.org—an intervention to help seniors make choices for their fourth quarter of life: results from the randomized clinical trial		
***PREPARE^b^* [34]**				Evidence obtained from at least one properly designed randomized controlled trial
	Howard et al [71]	Effect of an interactive website to engage patients in advance care planning in outpatient settings		
	Freytag et al [70]	Empowering older adults to discuss advance care planning during clinical visits: the PREPARE randomized trial		
	Lum et al [69]	Improving a full range of advance care planning behavior change and action domains: the PREPARE randomized trial		
	Zapata et al [68]	Feasibility of a video-based advance care planning website to facilitate group visits among diverse adults from a safety-net health system		
	Sudore et al [67]	Engaging diverse English- and Spanish-speaking older adults in advance care planning: the PREPARE randomized clinical trial		
	Sudore et al [2,66]	Effect of the PREPARE website vs an easy-to-read advance directive on advance care planning documentation and engagement among veterans		
	Cresswell et al [65]	Evaluation of an advance care planning web-based resource: applicability for cancer treatment patients		
	Ouchi et al [64]	Preparing older adults with serious illness to formulate their goals for medical care in the emergency department		
	Sudore et al [63]	A novel website to prepare diverse older adults for decision-making and advance care planning: a pilot study		
**The Letter project Advance Directive [55]**				Evidence obtained from at least one properly designed randomized controlled trial
	Periyakoil et al [72]	A randomized controlled trial comparing the letter project advance directive to traditional advance directive		

^a^Hierarchy of evidence from the National Health and Medical Research Council was assessed per tool. Level I: evidence obtained from a systematic review of all relevant randomized controlled trials; level II: evidence obtained from at least one properly designed randomized controlled trial; level III-1: evidence obtained from well-designed pseudorandomized controlled trials (alternate allocation or some other method); level III-2: evidence obtained from comparative studies with concurrent controls and allocation not randomized (cohort studies), case control studies, or interrupted time series with a control group; level III-3: evidence obtained from comparative studies with historical control, ≥2 single-arm studies, or interrupted time series without a parallel control group; level IV: evidence obtained from case series, either posttest or pretest, and posttest [25,26].

^b^PREPARE: PREPARE for Your Care.

## Discussion

### Principal Findings

This review included 30 ACP tools developed in North America, Europe, and Oceania. Most tools mention a clear aim (ie, to support reflection and communication, support people in making decisions, or support documenting decisions); however, only 7% (2/30) aimed to achieve all 3 aims. Of the 30 tools, 7 (23%) were developed in collaboration with health care professionals, but only 3 (10%) also involved end users. All tools, except 1, encouraged users to follow steps in a predetermined order to go through the ACP process. With regard to the ACP elements, almost all tools stimulated the user to explore personal values, goals, and preferences regarding future care; 40% (12/30) provided both information on ACP and its legal frameworks. Of the 3 tools, 2 (67%) also encouraged the user to appoint a proxy decision-maker. Most of the ACP tools had a good readability score; however, the quality of the content varied between 11 and 28 on the QUEST scale. Most of the included ACP tools had not been evaluated in a study.

We found great variety among the tools available on the web in terms of their aims, functionalities, approaches to addressing ACP, and quality. However, the included tools also shared important commonalities.

First, we found that many tools did not provide information about their development process. If they did, they involved health care professionals such as physicians, experts on end-of-life care, ethicists, or lawyers. End users were only involved in the development process of 10% (3/30) of tools, although this is highly recommended in the literature on developing web-based technologies [73-75]. Research on developing new technologies shows that the involvement of end users inevitably yields improvements in usability and quality and ensures that the tool is tailored to the needs of prospective end users [73-75].

Second, most tools stimulated the user to explore personal values, goals, and preferences regarding future care and the last days of life, which is the primary purpose of ACP [1,2], and most also encouraged the appointment of a proxy decision-maker and discussion with family and health care professionals. However, all but 1 tool in this review used an approach in which all users are encouraged to follow a predetermined path or step to go through the ACP process. ACP is a process of exploration; discussion; and recording of preferences, wishes, and decisions. How and when to best perform these aspects of ACP depends, among other things, on personal barriers, needs, preferences, and readiness [76,77]. Using steps in a predetermined order suggests that ACP is linear instead of an iterative process [1]. Moreover, using predetermined steps may not be appropriate for all users, as some may just want to explore possibilities without making decisions, whereas others, for instance, because of their illness, may prefer to focus on anticipatory decision-making [76,78].

Third, with regards to the quality of the content of the included tools, as rated by the QUEST score, we found that 20% (6/30) scored ≥20, of which 17% (1/6; PREPARE) scored a maximum of 28 points. All other tools had a medium to low quality score as they did not refer to any information sources and were not up to date; that is, they had not been updated in at least 5 years. Especially with regard to ACP, having evidence-based information that can be verified by the user is important as people use this information to plan and make health-related care decisions [79,80]. We also found that most of the included tools had not been evaluated in a study. Only the PREPARE tool had been evaluated for its ease of use, effectiveness, acceptability, and understandability. When people look for support in ACP, they may use tools that have a low or even nonexistent level of evidence regarding their usability and effectiveness [13-16,81].

### Strengths, Limitations, and Future Research

This is the first systematic review to provide an overview of interactive web-based ACP support tools available on the internet for the general population. Previous reviews have provided an overview of web-based ACP support tools that could be found in the peer-reviewed literature and emphasized the absence of an overview of those available in the gray literature [7,8]. Furthermore, our review is the first to assess the quality of the content of web-based ACP support tools [7,8]. This study had some limitations. First, as we limited our third search strategy (ie, search engine) to 150 *hits* per search combination, there is a possibility that some existing tools were not included in our review. However, we consider this unlikely, as our search was conducted systematically by 2 researchers using a broad range of search terms, and we searched more *hits* than recommended (recommended 100) by Godin et al [17]. We also consulted experts and asked them whether they knew of any other tools that we had not yet found. Furthermore, although we archived the tools when performing data synthesis using Archive.is, there is a possibility that new content or functionalities have been added to the tools included in our review or that new tools have been released since the search was conducted.

For future developments, users should be involved in aligning their preferences and needs with the content and functionalities of the tools. Involving users early in the development process can improve the usability of tools and increase their uptake [82]. In addition, we would recommend following a thorough design process using existing road maps when developing new ACP tools, rigorously evaluating through usability and effectiveness testing before deployment, and transparently reporting on development and evaluation. Second, the ACP content provided to users should be regularly updated and supported by sources; hence, we recommend that the content in ACP tools should be substantiated with the most recent scientific literature. Moreover, future research should focus on how ACP tools are used by the general population and how they can support ACP in the medical context; that is, between patients and health care professionals. Finally, it would be interesting to know how the general population would assess these tools.

### Conclusions

There are numerous interactive web-based ACP support tools that are publicly available, varying in terms of their characteristics, functionalities, readability, quality of content, and level of evidence. Most tools were not codeveloped with end users; were of low or medium quality; and, with a few exceptions, had not been evaluated in research. In the future, users should be involved in the development of ACP support tools, and their content should be substantiated by scientific evidence. In addition, we recommend that developers should follow a rigorous design process and evaluate the usability and effectiveness of tools before their deployment. Future research should focus on how tools are used by the general population and how they can support ACP in the medical context; that is, between patients and health care professionals.

## Appendix

Multimedia Appendix 1 Search terms for the web-based gray literature databases.

Multimedia Appendix 2 Search terms for the Google search engine.

Multimedia Appendix 3 Search terms for app stores.

Multimedia Appendix 4 Tools retrieved per search strategy.

Multimedia Appendix 5 Characteristics of the included interactive and web-based advance care planning support tools.

Multimedia Appendix 6 Functionalities of included interactive and web-based advance care planning support tools.

Multimedia Appendix 7 Advance care planning elements addressed in interactive web-based advance care planning support tools.

## Abbreviations

ACP: advance care planning
CEFR: Common European Framework of Reference for Languages
PREPARE: PREPARE for Your Care
PROSPERO: International Prospective Register of Systematic Reviews
QUEST: Quality Evaluation Scoring Tool
