# Supplementary material for: Publicly Available, Interactive Web-Based Tools to Support Advance Care Planning: Systematic Review
Source: J Med Internet Res. 2022 Apr 20;24(4):e33320. doi: 10.2196/33320 (PMC9069298; doi:10.2196/33320)
Supplement: Multimedia Appendix 1 [file jmir_v24i4e33320_app1.docx]

*Appendix 1 – search terms for online grey literature databases*

| 1 | "Advance care planning" AND ("Patient support tool" OR Tool OR "Conversation prompts" OR "Question Guide" OR "Tool kit" OR Website OR App OR application OR "Decision aid" OR "Conversation aid") |
| --- | --- |
| 2 | “Advance directive” AND ("Patient support tool" OR Tool OR "Conversation prompts" OR "Question Guide" OR "Tool kit" OR Website OR App OR application OR "Decision aid" OR "Conversation aid") |
| 3 | “Advance health care directive” AND ("Patient support tool" OR Tool OR "Conversation prompts" OR "Question Guide" OR "Tool kit" OR Website OR App OR application OR "Decision aid" OR "Conversation aid") |
| 4 | “End of life care” AND ("Patient support tool" OR Tool OR "Conversation prompts" OR "Question Guide" OR "Tool kit" OR Website OR App OR application OR "Decision aid" OR "Conversation aid") |
| 5 | “Shared decision making” AND ("Patient support tool" OR Tool OR "Conversation prompts" OR "Question Guide" OR "Tool kit" OR Website OR App OR application OR "Decision aid" OR "Conversation aid") |
| 6 | “Question prompts list” AND ("Patient support tool" OR Tool OR "Conversation prompts" OR "Question Guide" OR "Tool kit" OR Website OR App OR application OR "Decision aid" OR "Conversation aid") |
| 7 | “Living will” AND ("Patient support tool" OR Tool OR "Conversation prompts" OR "Question Guide" OR "Tool kit" OR Website OR App OR application OR "Decision aid" OR "Conversation aid") |
| 8 | “Personal directive” AND ("Patient support tool" OR Tool OR "Conversation prompts" OR "Question Guide" OR "Tool kit" OR Website OR App OR application OR "Decision aid" OR "Conversation aid") |
| 9 | “Advance decision” AND ("Patient support tool" OR Tool OR "Conversation prompts" OR "Question Guide" OR "Tool kit" OR Website OR App OR application OR "Decision aid" OR "Conversation aid") |
| 10 | "ACP" AND ("Patient support tool" OR Tool OR "Conversation prompts" OR "Question Guide" OR "Tool kit" OR Website OR App OR application OR "Decision aid" OR "Conversation aid") |
