# Supplementary material for: Publicly Available, Interactive Web-Based Tools to Support Advance Care Planning: Systematic Review
Source: J Med Internet Res. 2022 Apr 20;24(4):e33320. doi: 10.2196/33320 (PMC9069298; doi:10.2196/33320)
Supplement: Multimedia Appendix 2 [file jmir_v24i4e33320_app2.docx]

*Appendix 2 – search terms for search engine Google*

| 1 | "Advance care planning" AND ("Patient support tool" OR Tool OR Aid OR "Conversation prompts" OR "Question Guide" OR "Tool kit" OR Help OR Intervention OR Document OR Guide OR support OR "Decision aid" OR "Conversation aid") |
| --- | --- |
| 2 | "Advance directive" OR "Advance health care directive" AND ("Patient support tool" OR Tool OR Aid OR "Conversation prompts" OR "Question Guide" OR "Tool kit" OR Help OR Intervention OR Document OR Guide OR support OR "Decision aid" OR "Conversation aid") |
| 3 | "Shared decision making" AND ("Patient support tool" OR Tool OR Aid OR "Conversation prompts" OR "Question Guide" OR "Tool kit" OR Help OR Intervention OR Document OR Guide OR support OR "Decision aid" OR "Conversation aid") |
| 4 | "Question prompts list" AND ("Patient support tool" OR Tool OR Aid OR "Conversation prompts" OR "Question Guide" OR "Tool kit" OR Help OR Intervention OR Document OR Guide OR support OR "Decision aid" OR "Conversation aid") |
| 5 | "Living will" OR "Advance decision" AND ("Patient support tool" OR Tool OR Aid OR "Conversation prompts" OR "Question Guide" OR "Tool kit" OR Help OR Intervention OR Document OR Guide OR support OR "Decision aid" OR "Conversation aid") |
| 6 | "Advance care planning" AND start conversation with family OR start talk with family |
| 7 | "Advance care planning" AND start conversation with partner OR start talk with partner |
| 8 | "Advance care planning" AND help to talk |
| 9 | "Advance care planning" AND how to start |
| 10 | "Advance care planning" AND how to document OR how to do |
