# Supplementary material for: Publicly Available, Interactive Web-Based Tools to Support Advance Care Planning: Systematic Review
Source: J Med Internet Res. 2022 Apr 20;24(4):e33320. doi: 10.2196/33320 (PMC9069298; doi:10.2196/33320)
Supplement: Multimedia Appendix 3 [file jmir_v24i4e33320_app3.docx]

*Appendix 3 – search terms for app stores*

| 1 | "Advance care planning" |
| --- | --- |
| 2 | "Advance directive" |
| 3 | "Advance health care directive" |
| 4 | "End of life care" |
| 5 | "Shared decision making" |
| 6 | "Personal directive" |
| 7 | "Care for the future" |
| 8 | "End-of-life care planning" |
| 9 | "Decision aid" |
| 10 | "Conversation aid" |
