# Supplementary material for: Publicly Available, Interactive Web-Based Tools to Support Advance Care Planning: Systematic Review
Source: J Med Internet Res. 2022 Apr 20;24(4):e33320. doi: 10.2196/33320 (PMC9069298; doi:10.2196/33320)
Supplement: Multimedia Appendix 4 [file jmir_v24i4e33320_app4.docx]

*Appendix 4 – tools retrieved per search strategy*

|  | Tools found | After removing duplicates |
| --- | --- | --- |
| Grey databases | 133 | 27 |
| App stores | 37 | 7 |
| Google | 266 | 62 |
| Total | 436 | 96 |
