# Supplementary material for: Publicly Available, Interactive Web-Based Tools to Support Advance Care Planning: Systematic Review
Source: J Med Internet Res. 2022 Apr 20;24(4):e33320. doi: 10.2196/33320 (PMC9069298; doi:10.2196/33320)
Supplement: Multimedia Appendix 5 [file jmir_v24i4e33320_app5.docx]

*Characteristics of the included interactive, web-based advance care planning support tools.*

| **Name of the tool** | **Aim of the tool** | **Target group(s)** | **Language(s)** | **Format** | **Country of development** | **Developed by**  **(in year, (non-)profit)** | **Description on development** |
| --- | --- | --- | --- | --- | --- | --- | --- |
| ACDCare [28] | "The app guides you through the process to **fill out the ACD and then generates the official document** for South Australia and Northern Territory that you can carry with you and upload to MyHealthRecord." | Individuals and professionals | English | App | Australia | GP Partners Australia (non-profit) | No description available |
| Advance Care Planning: Should I Have Artificial Hydration and Nutrition? [35] | "This **decision aid** is for patients considering artificial hydration and nutrition if or when they are no longer able to take food or fluids by mouth.” | Individuals | English | Website | Canada | Healthwise and Alberta (2019, non-profit) | No description available |
| Advance Care Planning: Should I Receive CPR and Life Support? [36] | "This **decision aid** helps patients with serious or advanced illness decide whether or not to receive CPR and be put on a ventilator if heart or breathing stops.” | Individuals | English | Website | Canada | Healthwise and Alberta (2019, non-profit) | No description available |
| Advance Care Planning: Should I Stop Kidney Dialysis? [37] | "This **decision aid** helps patients with kidney failure who have been undergoing dialysis, and for whom kidney transplantation is not possible, decide whether to continue kidney dialysis, which will allow you to live longer, or stop kidney dialysis, which will allow death to occur naturally.” | Individuals | English | Website | Canada | Healthwise and Alberta (2019, non-profit) | No description available |
| Advance Care Planning: Should I Stop Treatment That Prolongs My Life? [38] | "This **decision aid** helps patients with serious or advanced illness decide whether to stop treatment that prolongs life and instead receive only hospice care, or to continue treatment that prolongs life.” | Individuals | English | Website | Canada | Healthwise and Alberta (2019, non-profit) | No description available |
| Be my voice [30] | No description about aim on the website | Individuals and professionals | English | Website | Australia | Capital Health Network (non-profit) | No description available |
| Beslishulp - Vroegtijdige zorgplanning [39] | No description about aim on the website | Individuals | Dutch | Website | Belgium | Centrum voor Evidence-Based Medicine (non-profit) | No description available |
| Cake [40] | "Our goal is to empower people to live in accordance with their **values** all the way to the end. This can only happen if we **know our end-of-life preferences** and **share** them with our loved ones." | Individuals | English | Online portal | United States | Cake (2015, profit) | No description available |
| Considering your own future health care [33] | No description about aim on the website | Individuals and professionals | English and Maori | Website | New Zealand | Health Quality & Safety Commission New Zealand (2018, non-profit) | No description available |
| Dementia Values and Priorities Tool [41] | No description about aim on the website | Individuals | English | Website | United States | Compassion & Choices (non-profit) | No description available |
| Dying to Talk [42] | "Dying to Talk encourages all Australians **to talk** about dying no matter your age, background or current health." | Individuals | English | Online portal | Australia | Palliative Care Australia (non-profit) | No description available |
| Everplans [43] | "Your Everplan helps you take control of your End-of-Life **planning process**." | Individuals | English | Online portal | United States | Everplans (2012, profit) | No description available |
| Five Wishes [44] | "**Completing** the Five Wishes **advance directive** can help you and your loved ones gain peace of mind around these difficult decisions." | Individuals | English | Online portal | United States | Aging with Dignity (1996, non-profit) | "Aging with Dignity founder J. Towey created Five Wishes with doctors, nurses, lawyers and other experts in end-of-life care." |
| Go Wish card game [45] | "The goal of the tool is **to facilitate discussions**, and for the end result of getting people to **think and talk** about positive values and goals for having the best end of life possible." | Individuals | English | Website | United States | CODA Alliance (2018, non-profit) | No description available |
| Lets Think Ahead – My ACP [46] | “This App will help you **develop your plan**, think about what’s important to you and help you be in control of your future” | Individuals | English | App | United Kingdom | University of the West of Scotland (non-profit) | No description available |
| My decisions [47] | "MyDecisions is a website which helps you plan ahead for your future treatment and care. This online service guides you through questions and scenarios **to help you think about the things** that are important to you." | Individuals | English | Online portal | United Kingdom | Compassion in Dying charity (non-profit) | No description available |
| My living voice [48] | No description about aim on the website | Individuals | English | Online portal | United States | Vital Decisions (profit) | No description available |
| My Living Will [31] | "My Living Will aims to help individuals and professionals **understand and take decisions** about care towards the end of life." | Individuals and professionals | English | Website | United Kingdom | My Living Will Charity (non-profit) | "A diverse group of senior healthcare professionals, senior lawyers and an ethicist have overseen My Living Will's creation of this website." |
| My Values [49] | "We specifically designed MyValues **to help address** an important problem – that we generally don't know what people would want when confronted by difficult life-and-death **decisions**." | Individuals | English | Online portal | Australia | Barwon Health (non-profit) | “This version of MyValues was created with valuable input from Sharyn Milnes, Nick Simpson, Jonathan Silverman and Julian Savulecu. The process was facilitated by Deb Porter with critical feedback from the Camperdown community group.” |
| MyDirectives [29] | "MyDirectives helps you **create your own digital advance care plan** or upload any advance directive, advance care plan or portable medical order you already have.” | Individuals | English | Online portal and App | United States | ADVault (non-profit) | No description available |
| MyWishes [50] | "We will help you; **write your last will & testament**, leave goodbye messages to be released after your death, make plans for your future health care, safeguard your digital legacy, publish your bucket list and document your funeral wishes." | Individuals | English | Online portal | United Kingdom | MyWishes (profit) | No description available |
| NVLivingWill [51] | NVLivingWill.com guides you in **completing your Living Will**, which is also known as an **Advance Directive**”. | Individuals | English, Spanish and Filipino | Online portal | United States | Nevada Center for Ethics & Health Policy (non-profit) | No description available |
| Oog in Oog [52] | “The Oog in Oog app has been developed **to provide guidance in the conversation** about the final phase of life” | Individuals and professionals | Dutch | App | The Netherlands | Bureau MORBidee (profit) | No description available |
| Plan your Life Span [53] | "This website will help you **plan** for health events such as hospitalizations, falls, and memory loss that may happen as people get older. This **planning** differs from end of life care and wills." | Individuals | English | Website | United States | Northwestern University (non-profit) | No description available |
| Planning for Your Future [54] | No description about aim on the website | Individuals | English | Website | Canada | People's Law School (non-profit) | No description available |
| PREPARE [34] | “PREPARE is a step-by-step program with video stories to help you: **have a voice** in YOUR medical care, **talk** with your doctors**, fill out an advance directive** form to put your wishes in writing.” | Individuals | English and Spanish | Website and Online portal | United States | University of California (2008, non-profit) | "PREPARE is the result of a collaboration of broad and talented group of people and organizations" and "Conducted **studies with diverse populations to further understand patients’ and caregivers’** needs for advance care planning". |
| Speak up [32] | "We provide a repository of resources and tools developed for professionals and patients/individuals to **assist them in making the appropriate decision** regarding their end of life care." | Individuals and professionals | English and French | Website | Canada | Canadian Hospice Palliative Care Association (non-profit) | No description available |
| The Letter project Advance Directive [55] | “To help, **empower and support** all adults to prepare for their future and take the initiative **to talk** to their doctors and their friends and family about what matters most to them at life's end.” | Individuals | English | Website | United States | Stanford Medicine (2015, non-profit) | "We conducted interviews and focus groups in multiple languages with people in the community **and talked to numerous patients and their family members** as well as health professionals" |
| Tijdig nadenken over het levenseinde [56] | "The digital **decision aid** helps you to form an opinion about your wishes regarding the last phase of your life. | Individuals | Dutch | Online portal | The Netherlands | NPV Zorg (non-profit) | No description available |
| Verken uw wensen voor zorg en behandeling [57] | "This **decision aid** helps you to **think** about wishes for care and treatment, and **to discuss** and **record them**. You can start this when you are still healthy, but also when you get older or get sick." | Individuals | Dutch | Website | The Netherlands | Dutch College of General Practitioners, Erasmus MC and thuisarts.nl (2020, non-profit) | “This decision aid was made by Erasmus MC in collaboration with: the Dutch College of General Practitioners, Kidney Patients Association, Netherlands NPVzorg.nl, Agora, LEVEL, University of Twente, and a sounding **board group of patients and relatives**.” |
