# Supplementary material for: Publicly Available, Interactive Web-Based Tools to Support Advance Care Planning: Systematic Review
Source: J Med Internet Res. 2022 Apr 20;24(4):e33320. doi: 10.2196/33320 (PMC9069298; doi:10.2196/33320)
Supplement: Multimedia Appendix 6 [file jmir_v24i4e33320_app6.docx]

Functionalities of included interactive, web-based advance care planning support tools.

| **Name of the tool** | Free of charge^a^ | No registration needed^b^ | Save and return^c^ | Possible to give input^d^ | Tailored^e^ | Feedback based on input^f^ | Input can be printed^g^ | Pre-determined path^h^ | Shows progress^i^ | Use of Video’s | Hyperlinks to other web pages | Text to speech option | Privacy policy mentioned | Data log analysis mentioned^j^ | Total |
| --- | --- | --- | --- | --- | --- | --- | --- | --- | --- | --- | --- | --- | --- | --- | --- |
| ACDCare [28] | x |  | x | x |  |  | x | x | x |  |  |  | x | x | 8 |
| Advance Care Planning: Should I Have Artificial Hydration and Nutrition? [35] | x | x |  | x |  |  | x | x | x |  | x |  | x | x | 9 |
| Advance Care Planning: Should I Receive CPR and Life Support? [36] | x | x |  | x |  |  | x | x | x |  | x |  | x | x | 9 |
| Advance Care Planning: Should I Stop Kidney Dialysis? [37] | x | x |  | x |  |  | x | x | x |  | x |  | x | x | 9 |
| Advance Care Planning: Should I Stop Treatment That Prolongs My Life? [38] | x | x |  | x |  |  | x | x | x |  | x |  | x | x | 9 |
| Be my voice [30] | x | x |  | x | x |  |  | x | x | x | x |  | x | x | 10 |
| Beslishulp - Vroegtijdige zorgplanning [39] | x | x |  | x | x |  | x | x | x |  | x |  |  | x | 9 |
| Cake [40] | x |  | x | x |  |  | x | x | x |  | x |  | x | x | 8 |
| Considering your own future health care [33] | x | x |  | x |  |  | x | x | x |  | x |  | x | x | 9 |
| Dementia Values and Priorities Tool [41] | x | x |  | x |  |  | x | x |  |  | x |  |  |  | 6 |
| Dying to Talk [42] | x | x | x | x |  |  | x | x | x |  | x |  | x | x | 10 |
| Everplans [43] |  |  | x | x |  |  | x | x | x |  | x |  | x | x | 8 |
| Five Wishes [44] |  |  | x | x |  |  | x | x | x |  |  |  | x | x | 7 |
| Go Wish card game [45] | x | x | x |  |  |  | x |  |  |  |  | x | x | x | 7 |
| Lets Think Ahead – My ACP [46] | x |  | x | x |  |  | x | x |  |  |  |  | x | x | 7 |
| My decisions [47] | x |  | x | x |  |  | x | x | x |  |  |  | x | x | 8 |
| My living voice [48] | x |  | x | x |  |  | x | x | x |  |  |  | x | x | 8 |
| My Living Will [31] | x | x | x | x |  |  | x | x | x | x |  |  | x | x | 10 |
| My Values [49] | x |  | x | x | x |  | x | x | x |  |  |  | x | x | 9 |
| MyDirectives [29] | x |  | x | x | x |  | x | x | x | x | x |  | x | x | 11 |
| MyWishes [50] | x |  | x | x |  |  | x | x |  | x | x |  | x | x | 9 |
| NVLivingWill [51] | x |  | x | x |  |  | x | x | x |  | x |  | x | x | 9 |
| Oog in Oog [52] |  |  | x | x |  |  | x | x | x |  |  |  | x | x | 6 |
| Plan your Life Span [53] | x | x | x | x |  |  | x | x | x | x | x |  |  |  | 9 |
| Planning for Your Future [54] | x | x |  | x |  |  |  | x |  | x | x |  | x | x | 8 |
| PREPARE [34] | x | x | x | x | x | x | x | x | x | x |  | x | x | x | 13 |
| Speak up [32] | x | x | x | x |  |  | x | x | x |  | x |  | x | x | 10 |
| The Letter project Advance Directive [55] | x | x |  | x |  |  | x | x |  |  |  |  | x | x | 7 |
| Tijdig nadenken over het levenseinde [56] | x |  | x | x |  |  | x | x | x | x | x |  |  |  | 8 |
| Verken uw wensen voor zorg en behandeling [57] | x | x |  | x |  |  | x | x | x | x | x | x | x | x | 11 |

^a^ Tool can be used without payment

^b^ Tool can be used without the need of registration or being logging in (using a personal logging and password)

^c^ People can save their process and return to continue their ACP process (via personal code/login system)

^d^ Responding to a question or statement in the tool for example via multiple-choice or typing text in an empty field

^e^ Content in the tool (ie information, questions, video’s etc.) are tailored based on the input of the user

^f^ Feedback such as to do’s or clarification on the impact of a choice are provided based on the input of the user

^g^ Generates document with input given by user

^h^ A “pre-determined path” was considered present if tools directed the user to follow a specific order. For example, when texts such as "you start at xx, and then move on to" or when indicators of such order were used such as numbers or steps (step 1, step 2, etc.)

^i^ The different steps that the user follows in the process, are shown in the tool itself

^j^ Tracking behaviour of the users in the tool is mentioned
