# Supplementary material for: Publicly Available, Interactive Web-Based Tools to Support Advance Care Planning: Systematic Review
Source: J Med Internet Res. 2022 Apr 20;24(4):e33320. doi: 10.2196/33320 (PMC9069298; doi:10.2196/33320)
Supplement: Multimedia Appendix 7 [file jmir_v24i4e33320_app7.docx]

*Advance care planning elements addressed in interactive, web-based advance care planning support tools.*

| Name of the tool | Information on: | | Addresses: | Stimulates to explore: | | | | | Encourage to: | | | | | | Total |
| --- | --- | --- | --- | --- | --- | --- | --- | --- | --- | --- | --- | --- | --- | --- | --- |
|  | ACP^a^ | legal frameworks^b^ | readiness and timing for ACP | personal values and goals | preferences regarding future care^c^ | Uncertainties and consequences^d^ | preferences regarding last days of life | possible PDM | appoint PDM | discuss with family^e^ | discuss with HCP^e^ | document^f^ | generate document^g^ | share document |  |
| ACDCare [28] |  |  |  | x | x |  |  | x | x | x | x | x | x | x | 9 |
| Advance Care Planning: Should I Have Artificial Hydration and Nutrition? [35] | x |  |  | x | x | x | x |  |  | x | x | x | x | x | 10 |
| Advance Care Planning: Should I Receive CPR and Life Support? [36] | x |  |  | x | x | x | x |  |  | x | x | x | x | x | 10 |
| Advance Care Planning: Should I Stop Kidney Dialysis? [37] | x |  |  | x | x | x | x |  |  | x | x | x | x | x | 10 |
| Advance Care Planning: Should I Stop Treatment That Prolongs My Life? [38] | x |  |  | x | x | x | x |  |  | x | x | x | x | x | 10 |
| Be my voice [30] | x | x | x |  |  |  |  | x | x | x | x | x |  |  | 8 |
| Beslishulp - Vroegtijdige zorgplanning [39] | x | x | x | x | x | x | x |  |  |  | x |  | x | x | 10 |
| Cake [40] |  |  |  | x | x | x | x | x | x | x | x | x | x | x | 11 |
| Considering your own future health care [33] | x |  |  | x | x | x | x | x | x | x | x | x | x | x | 12 |
| Dementia Values and Priorities Tool [41] |  |  |  | x | x | x | x |  |  | x | x |  | x | x | 8 |
| Dying to Talk [42] | x | x | x | x | x | x | x | x | x | x | x | x | x | x | 14 |
| Everplans [43] |  |  |  | x | x | x | x | x | x | x | x | x | x | x | 11 |
| Five Wishes [44] |  | x |  | x | x | x | x | x | x | x | x | x | x | x | 12 |
| Go Wish card game [45] |  |  | x | x | x |  | x |  |  | x |  | x | x | x | 8 |
| Lets Think Ahead – My ACP [46] | x |  | x | x | x | x | x | x | x | x | x | x | x | x | 13 |
| My decisions [47] |  | x | x | x | x | x | x | x | x | x | x | x | x | x | 13 |
| My living voice [48] |  |  |  | x | x |  | x | x | x | x | x | x | x | x | 10 |
| My Living Will [31] | x | x | x | x | x | x | x |  |  |  |  | x | x | x | 10 |
| My Values [49] | x | x | x | x | x | x |  | x | x | x | x | x | x | x | 13 |
| MyDirectives [29] | x | x | x | x | x | x | x | x | x | x | x | x | x | x | 14 |
| MyWishes [50] | x | x | x | x | x | x | x | x | x | x | x | x | x | x | 14 |
| NVLivingWill [51] | x |  |  |  | x | x | x | x | x | x | x | x | x | x | 11 |
| Oog in Oog [52] |  |  |  | x | x | x | x | x |  |  |  |  | x | x | 7 |
| Plan your Life Span [53] |  | x |  | x | x | x | x |  |  | x | x | x | x | x | 10 |
| Planning for Your Future [54] | x | x | x | x | x |  | x | x | x | x | x | x |  |  | 11 |
| PREPARE [34] | x | x | x | x | x | x | x | x | x | x | x | x | x | x | 14 |
| Speak up [32] | x | x | x | x | x | x | x | x | x | x | x | x | x | x | 14 |
| The Letter project Advance Directive [55] |  |  |  | x | x | x | x | x | x | x | x | x | x | x | 11 |
| Tijdig nadenken over het levenseinde [56] | x |  | x | x | x | x | x | x | x | x | x | x |  |  | 11 |
| Verken uw wensen voor zorg en behandeling [57] | x | x | x | x | x | x | x | x | x | x | x | x | x | x | 14 |

AD: advance directives

HCP: health care professional

PMD: proxy decision-maker, or health care representative, legal representative

^a^ Explanation what is ACP, why ACP, what are the goals, and limitations

^b^ Applicable law and regulatory framework w.r.t. ACP (including AD)

^c^ Preferences and goals regarding future (medical) care, future treatments and/or site of care excl. last days of life

^d^ Uncertainties and consequences of (hypothetical) serious illness scenarios

^e^ ACP communication such as what are preferences and goals regarding future (medical) care, future treatments and/or site of care, last days of life

^f^ To record and document ACP for example using an advance directive

^g^ Documentation of wishes, preferences and needs based on input of the user in the tool (not necessarily an AD, can also be a summary of the input of the user)
